# Efficacy of damage control surgery and staged endoscopic pancreatic ductal double stenting therapy for severe pancreatic head injury: a case report

**DOI:** 10.1186/s13256-021-02995-z

**Published:** 2021-08-13

**Authors:** Futoshi Nagashima, Satoshi Inoue, Daisaku Matsui, Yuki Bansyoutani, Rina Tokuda, Osamu Fuzisaki, Makoto Kobayashi

**Affiliations:** 1 Tajima Emergency and Critical Care Medical Center, Toyooka Public Hospital, 1094 Tobera, Toyooka, Hyogo 668-8501 Japan; 2 Inoue Clinic, 2-18-2 Togire, Nishi-ku, Fukuoka, 819-0032 Japan

**Keywords:** Pancreatic head injury, Endoscopic retrograde pancreatography (ERP), Damage control surgery (DCS), Endoscopic nasopancreatic drainage (ENPD), Endoscopic pancreatic stenting tube (EPST)

## Abstract

**Background:**

A high-grade pancreatic injury is a life-threatening injury that is associated with high mortality and morbidity. It is currently unclear which treatment strategy results in good clinical outcomes.

**Case presentation:**

A 23-year-old Japanese woman sustained severe injury in a motor vehicle accident. Abdominal computed tomography revealed severe pancreatic head injury with extravasation of contrast media. Since it was not possible to insert an endoscopic pancreatic stenting tube into the main pancreatic duct, damage control surgery was performed. On day 3, we could insert the endoscopic pancreatic stenting tube from the ampulla of Vater and an endoscopic nasopancreatic drainage tube in the distal pancreatic duct from the accessory ampulla before the second operation. Drainage tubes were placed around the pancreatic head in the second operation. The endoscopic nasopancreatic drainage tube tube was converted to endoscopic pancreatic stenting tube on day 9. On day 51, the patient was discharged on foot from our hospital without serious complications.

**Conclusion:**

Early and effective hemostasis, staged pancreatic duct drainage with stenting, and surgical external drainage around the pancreas in combination with an endoscopic procedure and damage control surgery were considered appropriate therapeutic strategy for high-grade pancreatic injury.

## Background

A high-grade pancreatic injury is rare, but it is a life-threatening condition that is associated with high mortality and morbidity due to serious complications, including leakage of pancreatic juice, hemorrhage, fistula, abscess, pancreatitis, and pseudoaneurysm [1].

Computed tomography (CT) imaging, magnetic resonance cholangiopancreatography (MRCP), and endoscopic retrograde cholangiopancreatography (ERCP) have been utilized to identify pancreatic duct injury. It was reported that the sensitivity and specificity of CT scans in the diagnosis of pancreatic duct injury were around 52–54% and 90–93%, respectively [2]. MRCP and ERCP have been shown to be effective as diagnostic tools for pancreatic duct injury; however, their application is limited based on reviews of case series [3]. The ERCP is able to both make a diagnosis and treat the patients, unlike the MRCP. However, the indication of ERCP, which is often invasive procedure for the patients, tends to be challenging owing to factors such as the general condition of the patients, technical difficulties, and complications after the procedure [3]. Grade III or IV pancreatic injury in the American Association for the Surgery of Trauma (AAST) grading system includes pancreatic duct injury in the pancreatic body and head. Since treatment failure following nonoperative management occurs with a fixed probability and a delay in surgical treatment leads to serious clinical outcomes, pancreatic resection and subsequent reconstructions have been performed in these high-grade pancreatic injuries [4]. However, various unwanted complications after these surgical procedures such as pancreatic fistula, intraabdominal bleeding, abscess, pseudocyst, pancreatitis, and pseudoaneurysm have been reported [5].

Hereby, we report a case of severe pancreatic head injury with pancreatic duct disruption where we were able to successfully treat the patient with no complication utilizing both damage control surgery (DCS) and staged endoscopic pancreatic ductal double stenting without pancreatic resection and complex reconstructions.

## Case presentation

**Fig. 1:**
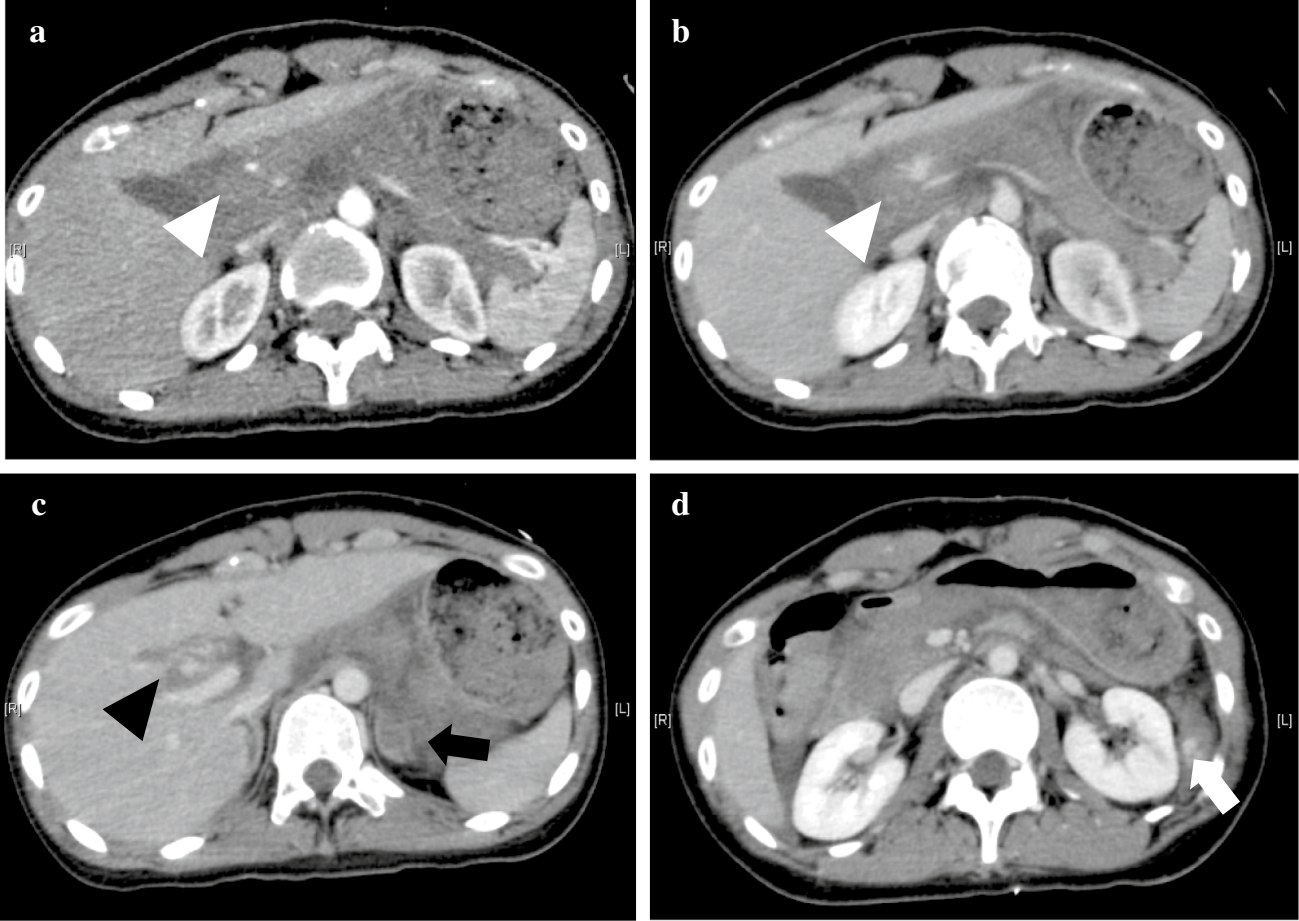
Computed tomography findings on admission. **a**, **b** Abdominal computed tomography scan revealing pancreatic head and body injury with hematoma and extravasation of contrast media (white arrowhead). **c**, **d** Abdominal CT showing liver injury (black arrowhead) and lower lobe of splenic injury (white arrow) with extravasation of contrast media and left adrenal injury (black arrow)

A 23-year-old Japanese woman sustained severe injury in head-on car collision to a utility pole when she was in a passenger seat. Her friend called 119, and the fire station simultaneously dispatched a doctor helicopter from our hospital. She was brought to our hospital by a doctor helicopter, where she underwent primary survey of trauma and initial fluid administration. She had no remarkable past medical history and family history, and no history of pregnancy or childbirth. There were no particulars in her social and environmental background that needed to be mentioned. She had been working in a hotel restaurant since the age of 22 years regarding her employment history. She has no history of alcohol or smoking. Her airway was patent; her respiratory rate was 26 breaths/minute, and blood oxygen saturation (SpO_2_) was 99% with an administration of oxygen at 10 L/minute. Her pulse rate and blood pressure were 75 beats/minute and 115/56 mmHg, respectively. Her consciousness level was 14 points (E3, V5, M6) according to the Glasgow Coma Scale, and no apparent paralysis of limbs was observed. No obvious trauma findings were observed on the head and face, except for a contusion on the right front forehead. There were no deviation of the trachea, subcutaneous emphysema, or jugular vein dilatation. She had tenderness in her posterior neck but no paralysis or sensory disturbance in her extremities. There were no obvious trauma findings on her chest and pelvis. Seat belt marks were noted on her right anterior chest and lower abdomen. Voluntary guarding and rebound tenderness were observed on her abdomen. She had marked spontaneous pain and tenderness in the upper lumbar spine, but had no abnormal neurological findings. There were no obvious abnormal findings on her extremities. Focused assessment sonography for trauma (FAST) revealed no hemoperitoneum. A whole-body contrast-enhanced CT was performed as her vital signs were stable. On abdominal CT, the head and body of her pancreas were remarkably swollen accompanied with hematoma, and an extravasation of contrast media from veins around the injured lesion was observed (Fig. 1a, b). Furthermore, the CT scan revealed a liver injury with extravasation of contrast media (Fig. 1c), injury of the lower lobe of spleen (Fig. 1d), left adrenal injury (Fig. 1c), fracture of the seventh cervical spinous process, and fracture of the first lumber vertebral body. The laboratory data of the patient on arrival at hospital are presented in Table 1.

**Table 1:** Laboratory data on admission

*Complete blood count*		
WBC	17,500	/μl
RBC	358	×10^4^/μl
Hb	10.7	g/dl
Ht	31.7	%
MCV	88.5	fl
MCH	33.8	pg
MCHC	33.8	%
Plt	21.7	×10^4^/μl
*Arterial blood gas*		
pH	7.39	
PCO_2_	35	mmHg
PO_2_	67	mmHg
HCO_3_^−^	21.2	mmol/l
BE	−3.2	mmol/l
Lac	3.5	mmol/l
*Chemistry*		
TP	5.8	g/dl
Alb	3.4	g/dl
BUN	11.6	mg/dl
Cre	0.88	mg/dl
eGFR	67	
Na	136	mmol/l
K	2.7	mmol/l
Cl	103	mmol/l
Ca	8.3	mg/dl
AST	114	IU/l
ALT	63	IU/l
LDH	492	IU/l
ALP	141	IU/l
T-Bil	0.4	mg/dl
CPK	106	IU/l
γ-GTP	9	IU/l
Glu	156	mg/dl
CRP	0.02	mg/dl
AMY	107	IU/l
P-AMY	65	IU/l
Lipase	226	U/l
*Coagulation*		
PT	69.6	%
PT-INR	1.25	
APTT	34.1	seconds
Fib	141.2	mg/dl
FDP	50	μg/ml
d-dimer	16.2	μg/ml

*Alb* albumin, *ALP* alkaline phosphatase, *ALT* alanine aminotransferase, *AMY* amylase, *APTT* activated partial thromboplastin time, *AST* aspartate aminotransferase, *BE* base excess, *BUN* blood urea nitrogen, *Ca* calcium, *Cl* chloride, *CPK* creatine phosphokinase, *Cre* creatinine, *CRP* C-reactive protein, *FDP* fibrin degradation product, *Fib* fibrinogen, *Hb* hemoglobin, *HCO*_3_^*−*^ bicarbonate, *Ht* hematocrit, *K* potassium, *Lac* lactate, *LDH* lactate dehydrogenase, *MCV* mean corpuscular volume, *MCH* mean corpuscular hemoglobin, *MCHC* mean corpuscular hemoglobin concentration, *Na* sodium, *P-AMY* pancreatic type amylase, *PCO*_*2*_ partial pressure of carbon dioxide, *Plt* platelets, *PO*_*2*_ partial pressure of oxygen, *PT* prothrombin time, *PT-INR* prothrombin time–international normalized ratio, *RBC* red blood cells, *T-Bil* total bilirubin, *TP* total protein, *WBC* white blood cells

**Fig. 2:**
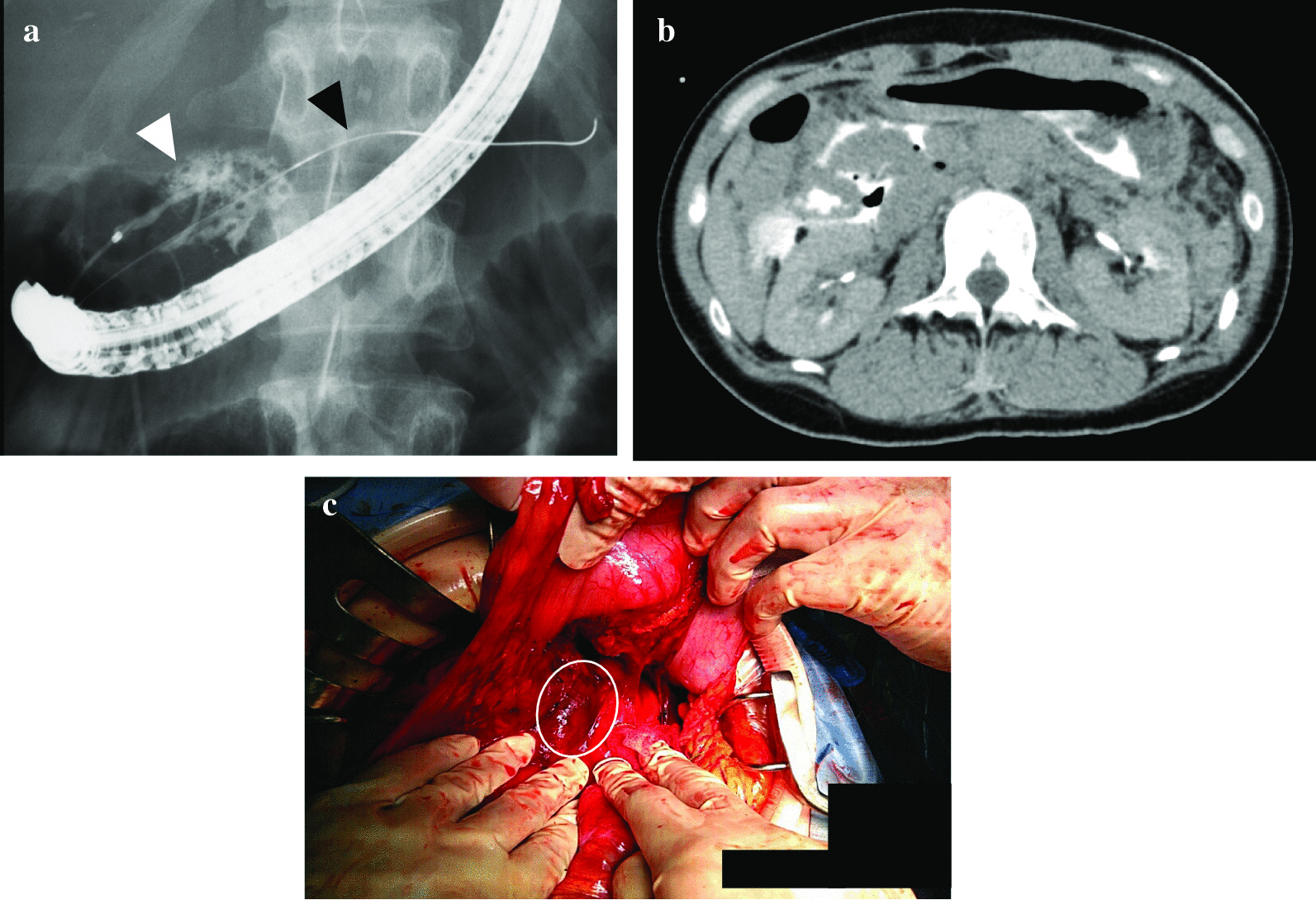
(**a**) Endoscopic retrograde pancreatography (ERP) findings, (**b**) CT findings after ERP, and (**c**) the findings during damage control surgery and views of the surgical site. Endoscopic retrograde pancreatography (ERP) findings in semi-prone position: Major leakage was found from the main pancreatic duct when contrast medium was injected from the ampulla of Vater (white arrowhead). Although the guidewire (black arrowhead) was able to be placed in the distal pancreatic duct, EPST and ENPD tube could not be cannulated to the distal duct over the injured sight. (**b**) Abdominal CT findings following ERP: abdominal CT revealing major leakage of contrast media and free air intraperitoneal and retroperitoneal space around the pancreas following ERP. (**c**) The findings during damage control surgery and views of the surgical site: a laceration with a diameter of approximately 2.5 cm and hemorrhage found on the cranial side of the pancreatic head by Kocher’s maneuver and entering omental bursa

We first performed an angiography to evaluate whether the patient had internal arterial hemorrhage. Since the main source of the bleeding was from vein around pancreatic injury, we could not perform transcatheter arterial embolization (TAE). Then, an endoscopic retrograde pancreatography (ERP) was performed to identify pancreatic duct injury. The catheter was inserted into the pancreatic duct from the ampulla of Vater, and a disruption of main pancreatic duct was observed when the contrast agent was injected through the catheter. We tried to cannulate endoscopic pancreatic stenting tube (EPST) both from the ampulla of Vater and the accessory ampulla to the distal pancreatic duct passing through the transected part; however, we were unable to insert the EPST. Although the guidewire was able to pass through the distal pancreatic duct, endoscopic nasopancreatic drainage (ENPD) tube could not be placed to the distal duct over the injured region (Fig. 2a). Abdominal CT revealed major leakage of contrast media and free air in the intraperitoneal and retroperitoneal space around the pancreas following ERP (Fig. 2b). Therefore, we decided to perform an emergency laparotomy.

During the crash laparotomy, major hematoma and moderate hemorrhage on the pancreatic head were observed. A laceration with a diameter of approximately 2.5 cm and diffuse complex contusion were found on the cranial side of the pancreatic head after the Kocher’s maneuver and entering omental bursa (Fig. 2c). Since continuous bleeding from the laceration and diffuse contusion were observed, we decided to perform damage control surgery (DCS). We did an abbreviated surgery in which gauze packing to control bleeding and leakage of pancreatic juice in the initial surgery was performed, followed by the second surgery where pancreatic resection in the injured region and drainage of pancreatic juice by the placement of pancreatic duct tube was performed. Then, reconstruction with pancreaticogastrostomy was performed in the third surgery. In the initial abbreviated surgery, peripancreatic gauze packing was performed, fitting bilateral surfaces of the laceration, and then temporary abdominal closure was performed with ABTHERA (total surgical time was 65 minutes). The patient was admitted to the intensive care unit (ICU) after the surgery.

**Fig. 3:**
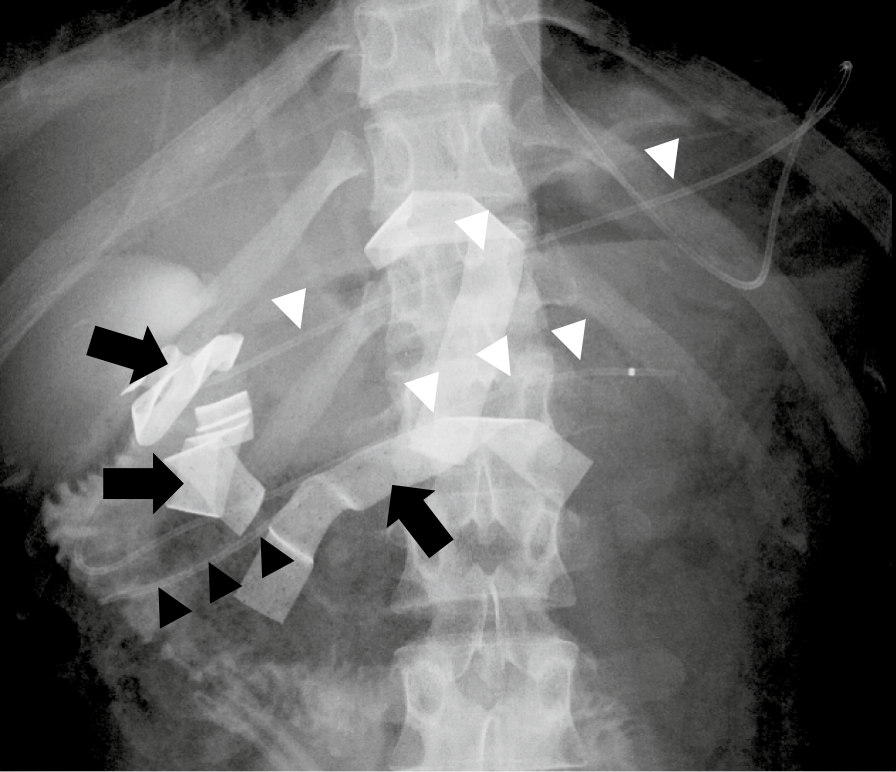
ERP findings on day 3. The placement of pancreatic endoscopic nasopancreatic drainage (ENPD) tube (white arrowhead) and endoscopic retrograde pancreatic drainage (ERPD) tube (black arrowhead) under ERP in a supine position on day 3. The black arrows point to the knitted strings in the packed gauzes. The white arrowheads represent ENPD tube. The black arrowheads represent ERPD tube

On day 3, we tried to perform ERP again before the second surgery. A 5 Fr ENPD tube was successfully placed in the distal pancreatic duct from accessory ampulla passing through the injured part (Fig. 3). Simultaneously, a 5 Fr EPST was inserted right next to the injured main pancreatic duct from the ampulla of Vater. Then, a second operation was performed. We confirmed that the bleeding from pancreatic injury had stopped, and the tissue damage around the pancreatic head was very partial and limited to a small area when the gauze was removed. The gauze packing played an important role in the control of both bleeding and leakage of pancreatic juice. The cholecystectomy was performed, and transcystic biliary drainage tube was placed in the common bile duct (CBD) from the cystic duct for external drainage of bile juice. The surgical drainage tube was placed on the posterior side of the pancreatic head and the anterior surface of the pancreatic laceration, and the abdominal wound was closed.

A peripheral venous line was inserted into the left upper extremity in the prehospital field, and a central venous (CV) line was inserted into the right internal jugular vein on admission. Midazolam was continuously administered from CV line as a sedative in the range of 0.5–1.0 mg/kg/hour, and fentanyl was continuously administered from CV line as an analgesic in the range of 1.0–1.5 μg/kg/hour according to the scale of sedation and analgesia during ventilation. Cefazolin (3 g/day) was administered via peripheral intravenous line until day 4. A proton pump inhibitor (60 mg/day) was administered as an antacid. From day 4, central venous nutrition (total parenteral nutrition, TPN) was administered from the CV line.

Her respiratory condition and circulatory status were stable after the second surgery. The tracheal tube was removed on day 4, and she was discharged from ICU to the general ward on day 7.

The patient was managed on nothing per os (NPO) until day 6, and then started oral liquid nutrition with limited fat in combination with TPN on day 7. On day 14, the patient’s nutrient intake was completely shifted from TPN to general hospital foods.

**Fig. 4:**
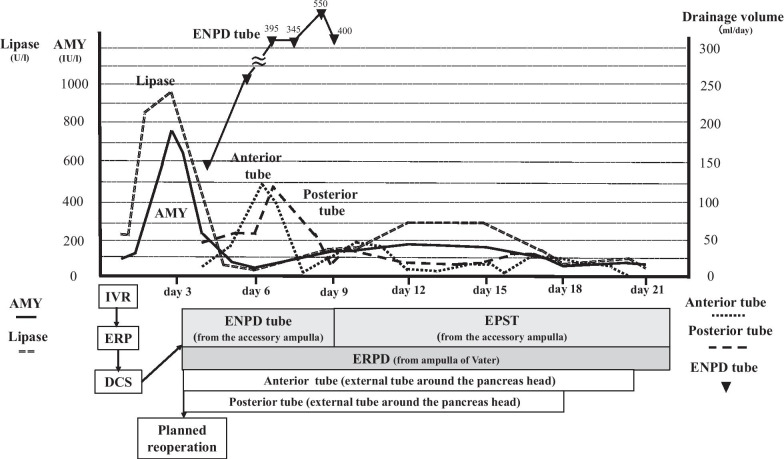
Changes in AMY and lipase level, drainage volume, and clinical course. The graph in the top half of Fig. 5 shows the change in AMY level and lipase in blood, and the change of drainage volume anterior and posterior tube around the pancreatic head, the change in drainage volume of the ENPD tube. The schematic diagram in the lower half of this figure shows the clinical course

**Fig. 5:**
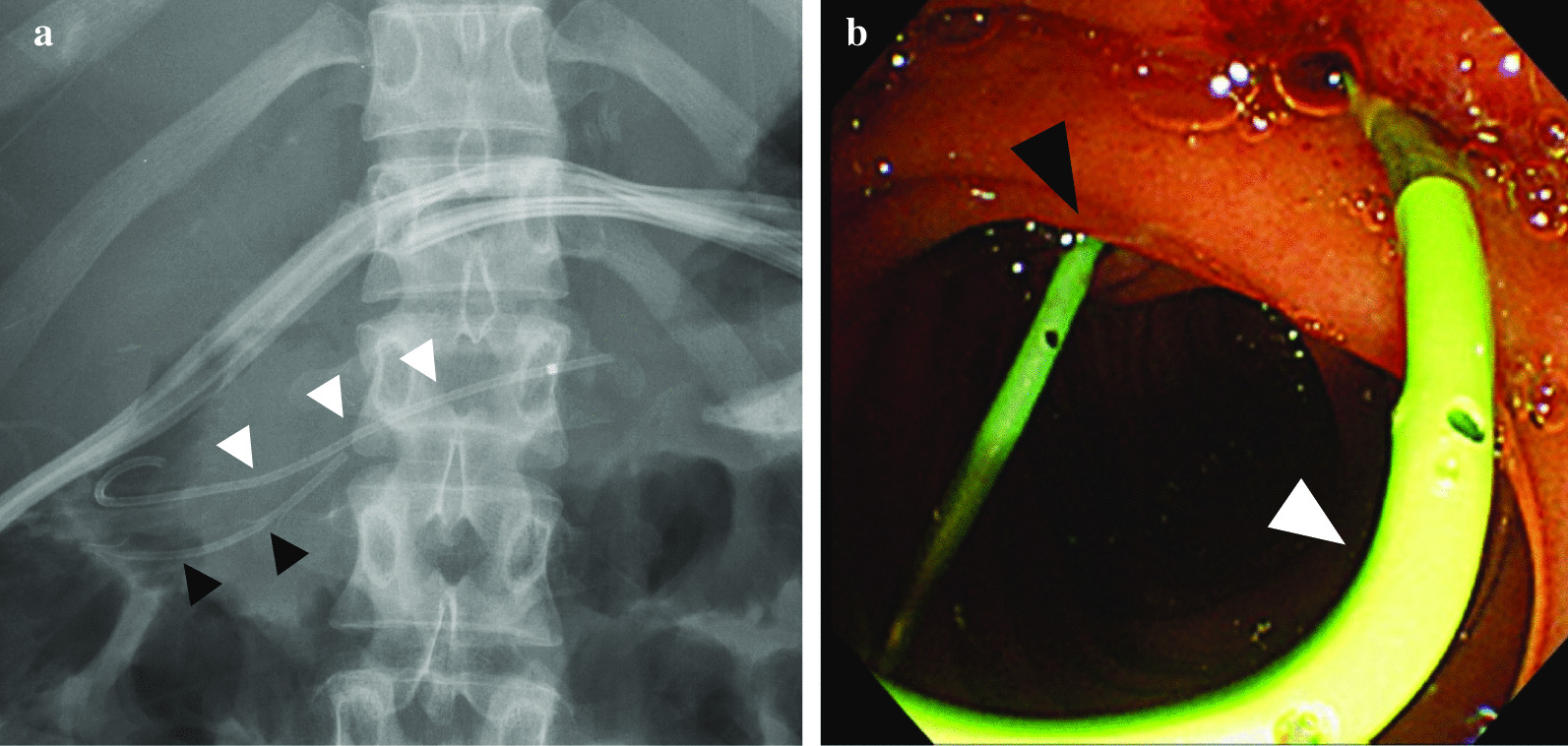
Abdominal X-ray and endoscopic findings on day 9. The double pancreatic duct stenting is shown on abdominal X-ray (**a**) and endoscopic findings (**b**). The ENPD tube was replaced by a 5 Fr endoscopic pancreatic stenting (EPS) (white arrowheads) for internal drainage on day 9. The black arrowheads show the ERPD tube that was placed on day 3

The amount of drainage of pancreatic fluid from the ENPD tube was approximately 400–500 ml/day. The amount of discharge from anterior and posterior surgical drainage tube around the pancreatic head tended to decrease as a maximum at about 125 ml/day on day 6 and 7, respectively (Fig. 4). The amylase value of those drainage fluid peaked at 2462 IU/l and 1094 IU/l on day 4, respectively, and then gradually decreased. The value of serum amylase became normal on day 5. The drainage of the pancreatic duct with ENPD was very effective, and it was replaced by a 5 Fr endoscopic pancreatic stenting tube (EPST) for internal drainage on day 9 (Fig. 5a, b). Fluid collection around the pancreatic head was not remarkable, and a pseudoaneurysm and an extravasation of contrast media in her abdominal cavity were no longer observed on CT on day 14. The posterior and anterior surgical external drainage tube around the pancreatic head were removed on day 18 and 20, respectively (Fig. 5). On day 38, 8.5 Fr of endoscopic biliary drainage (EBD) tube was placed in CBD from the ampulla of Vater for internal drainage in place of external drainage with C-tube.

Subsequently, her general condition improved, and the patient underwent rehabilitation for the first lumbar vertebral fracture. The patient’s medical condition fully recovered, and she was discharged on foot from our hospital on day 51.

Three months after discharge from the hospital, the bile duct stent and the EPST in the Wirsung duct were removed endoscopically. There were no abdominal symptoms after the removal of these tubes, and no elevation of biliary or pancreatic enzymes was observed. Five months after discharge, the EPST remaining in the Santorini duct was removed. She had no symptoms and no abnormal findings on blood tests and CT scan after the removal of the EPST. As a result, the follow-up was completed about 7 months after the injury.

## Discussion

This case report described a case of severe pancreatic head trauma that was successfully treated by a hybrid treatment strategy combining damage control surgery, staged endoscopic pancreatic duct stenting, and peripancreatic drainage, avoiding pancreaticoduodenectomy and complex reconstructive surgery without major complications.

Since the present case sustained pancreatic head injury with major pancreatic ductal injury with no arterial bleeding around the injured sight confirmed by CT and angiography, the ERP was performed immediately to evaluate the degree of pancreatic ductal injury. Firstly, we attempted to place endoscopic stenting or a drainage tube in the main pancreatic duct instead of surgical treatment, since complete disruption of the main pancreatic duct on the confluence of Wirsung duct and Santorini duct was detected on the ERP. However, nonoperative treatment with placing an ENPD tube or stents in the main pancreatic duct injury was not applicable on this patient. The first step in treatment of AAST grade IV pancreatic injuries is to determine whether the emergency surgical procedure is required. There were no significant differences in mortality, fistula formation, and hospital length of stay (LOS) between the operatively managed group and the nonoperatively managed group [1, 6]. However, it is reported that inappropriate treatment and a delay in surgical procedures after nonoperative management are likely to lead to an increase in morbidity and mortality [1]. In the present case, since nonoperative treatment with endoscopic pancreatic duct stenting resulted in failure, abbreviated life-saving surgery was performed, which was to pack around the pancreatic head with gauze without resection of the pancreas for temporary hemostasis and control of the pancreatic fluid. It was reported that there were no significant differences in mortality, fistula formation, intraabdominal abscess, and LOS between pancreatic resection group and non-resection group [7–9]. Peripancreatic gauze packing to fit both surfaces of laceration like we performed as DCS in this case was very effective because it allowed for the placement of the endoscopic stent and ENPD tube following DCS as well as hemostasis and decrease of the fatty saponification.

In our case, the efficacy of pancreatic fluid drainage by the ENPD tube placed in the main pancreatic duct through the Santorini duct from the accessory ampulla was significant because the amount of pancreatic fluid drainage from the ENPD tube continued to be approximately 400–500 ml/day. Therefore, the amount of surgical external drainage tube around the pancreatic head, which is placed during planned reoperation, remained very low. Furthermore, we thought that the ENPD tube drainage through the Santorini duct was not sufficient to control the pancreatic fluid leakage from the main duct injury in the confluence of the Wirsung duct and the Santorini duct. Therefore, the ERPD tube for the pancreatic duct was placed just next to the confluence from the ampulla of Vater. Consequently, both the ENPD tube and the ERPD tube greatly contributed to successful surgery without pancreatic resection and complex reconstructions requiring high level of skills and time-consuming procedures that may lead to unwanted complications.

With regard to the insertion of the ENPD tube, although a placement of ENPD tube in the main pancreatic duct as nonoperative management was impossible before the first surgery on day 1, it became possible to be placed on day 3. We attributed this successful placement of ENBD tube to pancreatic head gauze packing, which enabled the pancreas to restore its original shape for hemostasis in DCS. Although an initial ENPD tube insertion as nonoperative management failed, a repeated challenge of ENPD tube insertion in conjunction with surgery may have contributed to an effective pancreatic duct drainage leading to avoidance of unnecessary pancreatic resection, complex reconstructions, and postoperative complications. Furthermore, the cannulation into the distal main pancreatic duct from the accessory ampulla was much easier compared with the ampulla of Vater because of anatomical structure. If it was not possible to insert the ENPD tube into the distal main pancreatic duct through the injured lesion from the ampulla of Vater, it is worth trying to cannulate from the accessory ampulla. The ENPD tube was selected as the first device for drainage of the main pancreatic duct in our case. We firstly did not select the endoscopic direct stent for the pancreatic ductal drainage, and the ENPD was replaced with a stent when the ENPD drainage monitoring was no longer needed. We found that the monitoring of the amount of ENPD drainage was important to grasp the initial therapeutic effect on pancreatic injury.

The “SEALANTS” treatment was previously reported as a conservative approach to pancreatic duct disruption: somatostatin analogs, external drainage, alternative nutrition, antacids, nothing per os (NPO) status, total parenteral nutrition (TPN), and stenting of the pancreatic duct [10]. It was reported that there was no significant effect of somatostatin analogs on fistula formation rate and duration of fistula drainage [11]. In our case, all of the above treatments except for somatostatin analogs were performed. The amount of pancreatic fluid from external drainage was very low owing to effective ENPD tube drainage and placement of the stent. We thought that routine fistula prophylaxis with octreotide was not always needed if the ENPD tube or stent was securely placed into the main pancreatic duct for effective pancreatic fluid drainage.

Although most of the previous reports have compared the pancreaticoduodenectomy group with the non-resection group or the pancreatic resection group with the nonoperative treatment group for severe pancreatic head trauma of AAST grade IV or higher, it has been reported that it is difficult to make high-quality recommendations owing to the small number of cases and different conditions [1]. The hybrid treatment strategy performed in this case, combining damage control surgery, staged endoscopic pancreatic duct stenting, and peripancreatic drainage, is very unique compared with the above treatment methods in the following respects: (1) although endoscopic pancreatic duct stenting in the initial stage was difficult, it was successfully performed in a staged manner in combination with gauze packing in DCS, resulting in avoiding pancreaticoduodenectomy and complicated reconstructive surgery; and (2) double stenting of the main and accessory pancreatic ducts almost controlled leakage of pancreatic juice. As far as we have investigated, we could not find any report of treatment of severe pancreatic injury by this approach.

The most effective treatments for severe pancreatic head injury with major pancreatic duct injury should be performed in an early stage of injury. An appropriate hemostasis with DCS and staged pancreatic duct drainage with stenting and surgical external drainage around the injured pancreas should be considered first priority instead of pancreatic resection and complex reconstructions for successful treatment outcome of severe pancreatic injuries.

## Conclusion

The multimodal treatments combining endoscopic therapy and DCS, including early and appropriate hemostasis, staged pancreatic duct drainage with stenting, and surgical external drainage around the pancreas instead of pancreatic resection and complex reconstruction were considered feasible therapeutic strategy to pancreatic injury of AAST grade IV.

## Data Availability

Not applicable.

## Abbreviations

DCS: Damage control surgery
CT: Computed tomography
ERP: Endoscopic retrograde pancreatography
EPST: Endoscopic pancreatic stenting tube
ERPD: Endoscopic retrograde pancreatic drainage
ENPD: Endoscopic nasopancreatic drainage
MRCP: Magnetic resonance cholangiopancreatography
ERCP: Endoscopic retrograde cholangiopancreatography
AAST: American Association for the Surgery of Trauma
FAST: Focused assessment sonography for trauma
TAE: Transcatheter arterial embolization
ICU: Intensive care unit
CBD: Common bile duct
EBD: Endoscopic biliary drainage
LOS: Hospital length of stay
SEALANTS: Somatostatin analogs, external drainage, alternative nutrition, antacids, nothing per os (NPO) status, total parenteral nutrition (TPN), and stenting of the pancreatic duct
